# Surname‐Inferred andean ancestry is associated with child stature and limb lengths at high altitude in Peru, but not at sea level

**DOI:** 10.1002/ajhb.22725

**Published:** 2015-05-11

**Authors:** Emma Pomeroy, Jonathan C.K. Wells, Sanja Stanojevic, J. Jaime Miranda, Lorna G. Moore, Tim J. Cole, Jay T. Stock

**Affiliations:** ^1^ Newnham CollegeUniversity of CambridgeCambridgeUnited Kingdom; ^2^ Division of Biological AnthropologyDepartment of Archaeology and Anthropology, University of CambridgeUnited Kingdom; ^3^ Childhood Nutrition Research CentreUCL Institute of Child HealthUniversity College LondonLondonUnited Kingdom; ^4^ Division of Respiratory MedicineThe Hospital for Sick ChildrenTorontoOntarioCanada; ^5^ CRONICAS Center of Excellence in Chronic Diseases and Department of MedicineSchool of MedicineUniversidad Peruana Cayetano HerediaLimaPeru; ^6^ Department of Obstetrics/GynecologyUniversity of Colorado DenverAuroraColorado; ^7^ PopulationPolicy and Practice ProgrammeUCL Institute of Child HealthUniversity College LondonUnited Kingdom

## Abstract

**Objectives:**

Native Andean ancestry gives partial protection from reduced birthweight at high altitude in the Andes compared with European ancestry. Whether Andean ancestry is also associated with body proportions and greater postnatal body size at altitude is unknown. Therefore, we tested whether a greater proportion of Andean ancestry is associated with stature and body proportions among Peruvian children at high and low altitude.

**Methods:**

Height, head circumference, head‐trunk height, upper and lower limb lengths, and tibia, ulna, hand and foot lengths, were measured in 133 highland and 169 lowland children aged 6 months to 8.5 years. For highland and lowland groups separately, age‐sex‐adjusted anthropometry *z* scores were regressed on the number of indigenous parental surnames as a proxy for Andean ancestry, adjusting for potential confounders (maternal age and education, parity, altitude [highlands only]).

**Results:**

Among highland children, greater Andean ancestry was negatively associated with stature and tibia, ulna, and lower limb lengths, independent of negative associations with greater altitude for these measurements. Relationships were strongest for tibia length: each additional Andean surname or 1,000 m increase at altitude among highland children was associated with 0.18 and 0.65 *z* score decreases in tibia length, respectively. Anthropometry was not significantly associated with ancestry among lowland children.

**Conclusions:**

Greater Andean ancestry is associated with shorter stature and limb measurements at high but not low altitude. Gene‐environment interactions between high altitude and Andean ancestry may exacerbate the trade‐off between chest dimensions and stature that was proposed previously, though we could not test this directly. Am. J. Hum. Biol. 27:798–806, 2015. © 2015 The Authors American Journal of Human Biology Published by Wiley Periodicals, Inc.

Studies of responses to hypoxia among populations resident at high altitude for many generations provide a classic means of investigating both genetic adaptation and plasticity in development and growth (Baker and Little 1976; Beall, 2013; Frisancho, 1993). Native high‐altitude populations, including Andeans and Tibetans, are partially protected from the negative effects of hypoxia on birth weight compared with more recent migrants to altitude (Julian et al., 2007; Krampl et al., 2000; Zamudio et al., 1993). However, birth weight still continues to decrease with altitude among residents of Peru, the focus of this study, such that mean birth weight falls by ∼130 g per 1000 m increase in altitude (Mortola et al., 2000).

Despite their generally lower socioeconomic status (SES) compared with Andean residents of European ancestry, and this dose‐response “altitude‐penalty” on birth weight, neonates of Andean descent have higher birth weight than those of European ancestry at equivalent altitudes above ∼2500m. For example, Julian et al. reported that infants born in Bolivia to parents of Andean ancestry weighed on average 200–250 g more than those of European ancestry at the same altitude, controlling for gestational age and other known influences on birth weight (Julian et al., 2009; Vargas et al., 2007). The degree of protection from decreased birth weight correlates positively with the proportion of Andean ancestry (Julian et al., 2007; Soria et al., 2013), and paternal Andean ancestry exerts a stronger effect than maternal Andean ancestry by ∼80 g (Bennett et al., 2008).

Given that most studies investigating the relationship between ancestry and growth at altitude have been restricted to birth weight, there is a particular need to extend such research postnatally and to consider whether outcomes such as growth are affected. Understanding the relationship between Andean ancestry and growth, as measured by body size and proportions, at altitude can offer new insight into the developmental etiology of adaptations to hypoxia among native high‐altitude residents. Highland Andean children and adults are generally shorter than low‐altitude populations, with larger chests and shorter legs relative to the trunk (although see Frisancho et al., 1975a; Stinson and Frisancho, 1978), but some of these differences may be largely attributable to contrasts in healthcare, SES, and temperature which correlate negatively with altitude, rather than hypoxia per se (Frisancho et al., 1970; Greksa, 2006; Greksa et al., 1984; Niermeyer et al., 2009; Pawson and Huicho, 2010; Pawson et al., 2001). With decreasing temperature (increasing latitude), limbs are shorter relative to the trunk and distal limb segments become similarly shorter relative to proximal segments among modern and ancient human populations (Katzmarzyk and Leonard, 1998; Roberts, 1978; Trinkaus, 1981). However, the extent to which such variation in body proportions is genetically determined (Cowgill et al., 2012; Holliday, 1997) or is a direct effect of ambient temperature on growth (Serrat et al., 2008) remains uncertain.

Several hypotheses are proposed concerning the relationship between Andean ancestry and postnatal body size and proportions. Hypothesis 1 is that, other factors being equal, individuals with greater Andean ancestry grow taller and are heavier postnatally as well as prenatally at altitude as a result of genetic adaptations to hypoxia. Recent studies have detected signatures of natural selection among Andeans in genes associated with the hypoxia‐inducible factor (HIF) pathway, which is involved in tissue oxygen homeostasis (Bigham et al., 2010, 2009). However, the phenotypic effects of these genotypes are uncertain, although progress is being made in this regard (Bigham et al., 2014). Andeans do not have major genetic variants that elevate arterial oxygen saturation, unlike Tibetans (Beall et al., 1999, 1994), and variants of the genes *EGLN1* and *EPAS1*, which show evidence of positive selection in highland Andeans, are not associated with blood hemoglobin concentration (Bigham et al., 2013). Alternatively, numerous studies show that highland Andean children have larger chests and lung volumes for their age and/or height compared with non‐Andeans at high altitude (Beall, 1982; Brutsaert et al., 1999; Frisancho et al., 1975a; Hoff, 1973; Mueller et al., 1978b; Palomino et al., 1978; Stinson, 1980, 1982), although Tibetans and Europeans are also reported to have increased chest and/or lung dimensions at high altitude compared with their low‐altitude counterparts (Beall 1982; DeGraff et al. 1970; Droma et al. 1991; Greksa 1986; Greksa 1988; Greksa and Haas 1982; Greksa et al. 1988).

While larger chest/lungs could increase arterial oxygenation and thus reduce hypoxic constraints on growth (consistent with Hypothesis 1), others have proposed that the greater acceleration of chest growth among highland Andeans is compensated for by reduced height (Frisancho, 2013; Frisancho et al., 1975b; Mueller et al., 1978a; Pawson and Huicho, 2010). Hypothesis 2 is therefore that Andean ancestry is associated with altered postnatal growth patterns, including shorter stature. Various studies suggest that under conditions of environmental stress (e.g., hypoxia, malnutrition) growth is prioritized in some parts of the body, typically the brain (Barbiro‐Michaely et al., 2007; Barker, 1998; Giussani, 2011; Wells, 2013) at the particular expense of lower limb and distal limb segment (tibia) length and, to a lesser extent, trunk length (Bailey and Hu, 2002; Bailey et al., 2007; Bogin et al., 2002; Gunnell et al., 1998; Lampl et al., 2003; Pomeroy et al., 2012; Pomeroy et al., 2013; Whitley et al., 2008). Thus, if brain and chest size are prioritized over stature among children of Andean ancestry, we might also anticipate that lower limb and especially tibia length experience the greatest reductions in growth, altering relative trunk and limb proportions.

Two variants of Hypothesis 2 may also be proposed. Hypothesis 2a is that patterns of chest, stature, and limb proportions associated with Andean ancestry are genetic adaptations to hypoxia and expressed regardless of altitude. Hypothesis 2b is that the Andean growth pattern results from gene‐environment interactions whereby greater chest size has a genetic basis, but is facultatively expressed during development at high altitude. Previous studies suggest that accelerated Andean chest development is conditional upon high‐altitude exposure and ancestry: children of European ancestry also develop relatively larger chests at high altitude (Greksa, 1986, 1988; Greksa and Haas, 1982; Greksa et al., 1988), but to a lesser extent than Andeans (Brutsaert et al., 1999; Greksa, 1986, 1988; Greksa et al., 1988; Palomino et al., 1978; Stinson, 1982). If the highland Andean body plan is contingent on hypoxia exposure, Andean ancestry would be expected to be associated with stature and limb proportions among highland but not lowland children. At low altitude, no increase in chest size would be favored; hence, limb growth would not be sacrificed.

The purpose of this study was to examine the relationship between native Andean ancestry (inferred from surname analysis) and the size and proportions of the head, trunk, limbs, and limb segments among highland and lowland Peruvian children. We aimed to investigate which of the hypotheses outlined best accounts for any variation in postnatal body size and proportions associated with Andean ancestry by evaluating the following predictions:

Hypothesis 1. If genetic adaptation to hypoxia benefits overall growth at altitude, highland children with a greater proportion of Andean ancestry will have a taller stature and longer limbs;

Hypothesis 2a. If genetic adaptation to hypoxia among native Andeans favors a larger chest at the expense of stature and limb length, a higher proportion of Andean ancestry will be associated with shorter stature and limbs regardless of altitude;

Hypothesis 2b. If genetic adaptation to hypoxia among native Andeans favors a larger chest at the expense of stature and limb length only on exposure to hypoxia, a greater degree of Andean ancestry will be associated with shorter stature and limbs among highland children only.

## Methods

The sample is part of that from a larger study of body size and proportions among Peruvian children (Pomeroy et al., 2012). A convenience sample of 447 children was recruited from two populations. The first population was the “*pueblo joven*” (shanty town) of Pampas de San Juan de Miraflores on the south side of Peru's capital, Lima (latitude −12.0, longitude −77.0, altitude 140 m). The settlement was unplanned and comprised of migrants predominantly from highland Peru. Although it remains a relatively low SES community, many houses are now more substantially constructed and are connected to water and sewage systems (Checkley et al., 2004; Masterson Creber et al., 2010; Miranda et al.. 2009; Sterling et al., 2012). There are also schools and health posts in the settlement. The highland sample came from small, rural communities in the Vinchos and Santillana districts of Peru's Ayacucho Region (latitude −13.2, longitude −74.2 for Ayacucho city). Communities were located at 3,100–4,400 m altitude, and comprised mainly of subsistence agropastoralists (Masterson Creber et al., 2010; Miranda et al., 2009).

Participants of the original study were aged 6 months to 14 years, falling within specific target age groups (Pomeroy et al., 2012). Date of birth was confirmed using national identity documents, birth record cards, or school records. Participants had been born and raised in their study region, and did not have any medical conditions, which might compromise growth aside from general undernutrition. A maximum of one child per household participated in the study on a voluntary basis. A parent or guardian provided written informed consent, and participants aged 6 years or over gave verbal or written assent. The study was approved by the Institutional Ethics Committee at the Universidad Peruana Cayetano Heredia, Lima, and the Health Directorate for Ayacucho Region (Dirección Régional de Salud Ayacucho, DIRESA).

Anthropometry was measured by one trained observer (EP) following standard techniques (Cameron, 2004; Lohman et al., 1988). Standing and head‐trunk (sitting) heights were measured to the nearest mm with a Seca Leicester Height Measure, or as crown‐heel and crown‐rump length in children aged less than 2 years using a Rollametre (Dunmow, UK). Total lower limb length was calculated by subtracting head‐trunk from standing height (or crown‐rump from crown‐heel length). Tibia, ulna, upper arm, hand, and foot lengths were measured to the nearest mm using sliding callipers, and total upper limb length calculated by summing ulna and upper arm length. Head circumference was measured using a 15 mm‐wide nonstretch fiberglass tape (Hoechtmass, Germany). Intraobserver error on adult subjects gave a technical error of measurement below 1% and a coefficient of reliability (*R*) of at least 0.97 for all measurements, which were judged to be within acceptable limits (Ulijaszek and Kerr, 1999).

Parental surnames were used as a proxy for ancestry as in various studies in the Andes and elsewhere (Azcorra et al., 2013; Chakraborty et al., 1989; Colantonio et al., 2003; Relethford, 1995). Peruvians inherit the paternal surname of their mother and father, giving each individual two surnames, and they do not change their surnames at marriage. Therefore, the linguistic origin of a participant's parents' surnames (giving 4 surnames for analysis) can be used to infer the proportion of indigenous ancestry of that individual. While an imperfect marker (Brutsaert, 2001; Greksa, 1992), this approach has been used successfully in numerous studies of Andean populations that have aimed to relate birth characteristics to ancestry (Bennett et al., 2008; Gonzales et al., 1996; Julian et al., 2007; Mueller et al., 1978a; Soria et al., 2013) and the association between native ancestry and surname origin in the Andes has been previously validated (Chakraborty et al., 1989). The four parental surnames of each individual were classified as “indigenous,” “mestizo,” (mixed) or “foreign” based on their linguistic origin using a surname dictionary complied by one of the authors (LGM).

Sex‐age‐specific internal *z* scores were calculated for anthropometry in the combined lowland and highland sample after fitting centiles using the LMS method (Cole and Green, 1992), and analyses used these *z* scores throughout. For the highland and lowland groups, anthropometry z scores were regressed separately on the number of indigenous surnames (entered as a continuous variable, range 0–4) and potential confounding variables using a backwards stepwise model where potential confounders with *P* < 0.1 were retained. Potential confounders were altitude (highland sample only), maternal education (an aggregate marker of SES, e.g., Jansen et al., 2009; Kramer et al., 2000; Raum et al., 2001; van den Berg et al., 2013; which was used as such in previous study of Andean surnames and growth: Bennett et al., 2008), maternal age and birth order. These variables were included as they have previously been shown to relate to child growth and/or body proportions (Dasgupta et al., 2008; Hatton and Martin, 2010; Lawson and Mace, 2008; Li et al., 2007; Li and Power, 2004; Semba et al., 2008; Whitley et al., 2008), and because they are correlated with the number of indigenous parental surnames in our sample. Indigenous surnames increase in frequency with altitude in the Andes because European migrants arriving from the 16^th^ century onwards generally settled at low to mid altitudes, and perhaps because indigenous Andeans tend to adopt more Spanish‐sounding surnames as they acculturate (Schull and Rothhammer, 1977), while SES and access to education decrease at higher altitude (Niermeyer et al., 2009; Rivera‐Ch et al., 2008).

As patterns of growth are complex and transient during puberty, analyses were restricted to children aged 6 months to 8.5 years (lowland *n* = 169, highland *n* = 133). Although pubertal onset was not assessed directly, the vast majority of children aged below 8.5 years were pre‐pubertal in a similar low SES population in the Americas (Wilson et al., 2011). Analyses were restricted to individuals who had all four parental surnames recorded, as well as all potential confounding variables for the regression models (exclusions are detailed in Supporting Information Table S1). Within each sample, t‐tests or chi‐square tests as appropriate confirmed that there were no significant differences between those with and without full surname data in terms of altitude, maternal age or education, or anthropometry *z* scores (height, head circumference, sitting height, and tibia length: *P* > 0.05, results not shown). Analyses of relative total limb, ulna, tibia hand and foot lengths were performed by adjusting for head‐trunk height z score in the regression model. Analyses were conducted in SPSS v.21, with *P* values below 0.05 considered significant.

## Results

**Figure 1:**
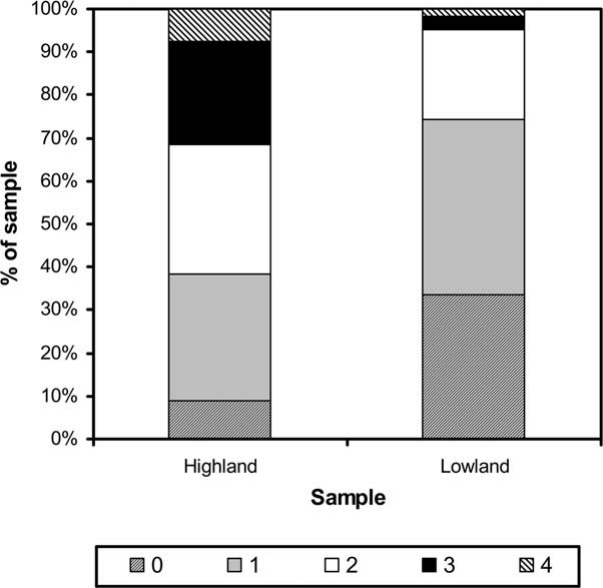
Numbers of indigenous parental surnames among highland and lowland Peruvian children in the sample.

Tables 1, 2, 3 summarize the characteristics and anthropometry of the highland and lowland samples. Samples were approximately evenly divided between males and females. Most children had a mix of indigenous and mestizo or foreign surnames, and foreign surnames were more common in the lowland sample (Fig. 1). Seven percent of highland children had four indigenous surnames, while 9% had none, compared with 2 and 34% of lowland children respectively (highland‐lowland difference by *χ* ^2^: *P* < 0.001). Only 6% of highland mothers had secondary education or higher, while 19% had not attended school. In contrast all lowland mothers had some level of education and 77% had complete secondary education or higher (*χ* ^2^: *P* < 0.001). Half of lowland children were first‐born, while only 30% of highland children were first‐born and one‐third were the fourth child or later born (*χ* ^2^: *P* < 0.001). Highland children had lower mean anthropometry *z* scores for all measurements than lowland children (Table 3: highland‐lowland differences have *P* < 0.001 for all measurements using t‐test). Further details of these samples and differences in anthropometry were reported previously (Pomeroy et al., 2012, 2013, 2014).

**Table 1:** Age and sex distribution of the sample

Age group (years)	Lowland			Highland		
	Female (*n*)	Male (*n*)	Total (*n*)	Female (*n*)	Male (*n*)	Total (*n*)
0.5–1.49	14	15	29	13	13	26
1.5–2.49	16	14	30	7	10	17
2.5–3.49	13	14	27	15	11	26
3.5–4.49	13	12	25	13	14	27
5.5–6.49	16	15	31	9	13	22
7.5–8.49	14	13	27	9	6	15
Total	86	83	169	66	67	133

**Table 2:** Summary statistics on parental surnames, altitude, birth order, maternal education, maternal age, and offspring anthropometry z scores for the highland and lowland study samples

Variables	Highland			Lowland		
Categorical	*n*	%	Regression category^a^	*n*	%	Regression category
No. indigenous parental surnames						
0	12	9		56	33	
1	39	29		68	41	
2	40	30		35	21	
3	32	24		5	3	
4	10	8		3	2	
Birth order						
1	40	30		84	50	
2	29	22		51	31	
3	19	14		20	12	
4+	45	34		12	7	
Maternal education						
None, illiterate	23	17	1	0		1
None, literate	2	2	1	0		1
Primary, incomplete	70	53	2	3	2	1
Primary, complete	30	22	3	6	4	1
Secondary, incomplete	4	3	3	29	17	1
Secondary, complete	3	2	3	68	41	2
Postsecondary, incomplete	0	0	3	25	15	3
Postsecondary, complete	1	1	3	36	22	4
Continuous	Mean	SD		Mean	SD	
Maternal age at birth of child (yrs)	25.1	7.6		26.4	6.3	
	Median	IQR				
Altitude measured (m)	3,730	3,320–3,830		–	–	

a Regression Category" indicates how original categories were recoded for the purpose of the regression model: categories with small numbers of cases were combined.

**Table 3:** Summary statistics for child anthropometry, giving mean measurements at age 4 years by sex, and mean internal age‐sex adjusted z scores by site

Variable	Measurement (in cm) at age 4 years: pooled sample^a^				Sex‐age specific *z* scores					
	Males		Females		Highland			Lowland		
	Mean	SD^b^	Mean	SD	Mean	SD	*n*	Mean	SD	*n*
Head circumference	49.5	1.3	48.9	1.4	−0.4	0.8	125	0.3	1.0	165
Stature	98.1	5.0	97.2	5.2	−0.8	0.7	133	0.7	0.7	167
Head‐trunk height	57.9	2.5	57.3	2.7	−0.6	0.8	131	0.5	0.9	167
Total upper limb length	33.7	2.3	33.4	2.5	−0.9	0.7	106	0.6	0.7	154
Ulna length	14.7	1.0	14.5	1.1	−0.9	0.7	108	0.6	0.7	163
Hand length	11.3	0.7	11.3	0.7	−0.8	0.7	103	0.5	0.8	156
Total lower limb length	40.3	3.0	39.9	3.0	−0.8	0.8	131	0.7	0.7	167
Tibia length	19.4	1.7	19.3	1.6	−0.9	0.7	116	0.7	0.6	162
Foot length	15.5	1.0	15.3	1.0	−0.7	0.7	116	0.6	0.8	163

a Derived from LMS model (Cole and Green, 1992) for the full dataset.

b SD: standard deviation.

Among highland children, more indigenous parental surnames were associated with shorter tibia length, relative tibia length, stature, lower limb length, ulna length and relative lower limb length (in order of decreasing regression coefficients for number of indigenous parental surnames: Table 4). Increased altitude was similarly associated with shorter measurement *z* scores except for head‐trunk height, head circumference, and foot length. An additional indigenous surname was associated with a 0.18 *z*‐score decrease in tibia length, while 1,000 m increase in altitude was associated with a 0.65 *z*‐score decrease in tibia length. This compares with 0.14 and 0.62 *z*‐score decreases in stature with the same increases in indigenous surnames and altitude respectively, clearly very similar. Full details of the regression models including potential confounders included in each model are provided in Supporting Information Table S2.

**Table 4:** Results of regression models of anthropometry z scores on the number of indigenous parental surnames among highland and lowland Peruvian children, adjusting for potential confounding variables

Measurement *z* score	Highland					Lowland	
	Altitude (km)		Number of indigenous parental surnames			Number of indigenous parental surnames	
	*B*	*P*	*B*	Standard error (B)	*P*	*B*	*P*
Tibia length	−**0.65**	**<0.001**	−**0.18**	**0.05**	**0.002**	0.03	0.6
Relative tibia length	−**0.49**	**0.001**	−**0.13**	**0.05**	**0.005**	−0.004	0.9
Lower limb length	−**0.66**	**0.001**	−**0.16**	**0.06**	**0.01**	−0.02	0.7
Stature	−**0.62**	**0.001**	−**0.14**	**0.06**	**0.01**	0.04	0.5
Ulna length	−**0.72**	**<0.001**	−**0.12**	**0.05**	**0.03**	0.01	0.9
Relative lower limb length	−**0.57**	**0.002**	−**0.12**	**0.06**	**0.04**	−0.04	0.5
Head‐trunk height		ns	−0.12	0.06	0.06	0.08	0.3
Head circumference		ns	−0.11	0.07	0.08	−0.03	0.7
Relative ulna length	−**0.49**	**0.001**	−0.08	0.04	0.09	−0.02	0.7
Upper limb length	−**0.61**	**0.001**	−0.09	0.06	0.1	0.02	0.7
Hand length		ns	−0.09	0.06	0.2	−0.03	0.7
Foot length	−0.37	0.07	−0.09	0.06	0.2	−0.02	0.7
Relative upper limb length	−**0.40**	**0.003**	−0.05	0.05	0.5	−0.01	0.8

**Bold** indicates *P* < 0.05. Blank cells denote variable excluded from model as *P* < 0.1.

See Supporting Information Tables S2 and S3 for details of confounders for highland and lowland samples respectively. Variables are ordered in the table by *P* values for number of indigenous surnames among highland children.

Among lowland children, anthropometry *z* scores were not associated with the number of indigenous parental surnames (Table 4, full model details in Supporting Information Table S3).

## Discussion

Our results indicate that a greater number of indigenous parental surnames was associated with reduced stature and the lengths of the tibia, lower limb and ulna, but not head circumference or hand and foot lengths, among highland children after adjusting for relevant factors including altitude. The strongest associations were observed with tibia length. In contrast, there were no such associations among lowland children. Among highland children, anthropometry was negatively associated with higher altitude independent of the number of indigenous surnames. We considered that these results were most consistent with Hypothesis 2b; namely, that accelerated chest growth at altitude, dependent both on hypoxia exposure and augmented by Andean ancestry, results in reduced growth elsewhere in the body (see Introduction). While this is consistent with what others have proposed (Frisancho, 2013; Frisancho et al., 1975b; Mueller et al., 1978a; Pawson and Huicho, 2010), we lacked chest size data to test this directly. The strongest associations with ancestry among highland children were for tibia length, consistent with previous studies that suggest that tibia length is particularly sensitive to environmental conditions (see Introduction). This pattern of results supports the interpretation that ancestry‐related differences in body dimensions result from a growth trade‐off at altitude. The results were not consistent with an adaptation among native Andeans that promotes greater height and longer limbs at altitude (Hypothesis 1). The lack of association between ancestry and height or body proportions among lowland children does not suggest that reduced stature and limb lengths is a general characteristic of Andeans regardless of altitude (Hypothesis 2a).

If accelerated chest growth among high‐altitude Andeans represents an adaptation to reduce tissue hypoxia (Frisancho, 2013; Frisancho et al., 1975b; Mueller et al., 1978a; Pawson and Huicho, 2010), it does not seem to lead to increased growth and stature. Any advantage in terms of body size may only be achieved once growth is complete, since growth velocity has been noted to be slower at high altitude (Frisancho, 1976). Some studies indicate that increased chest size relative to stature is more marked in children than adults (Hoff, 1973; Mueller et al., 1978a,1978b; Palomino et al., 1978), suggesting that height “catches up” with chest growth later in development. Alternatively, the larger chest dimensions of highland Andeans are associated with improved lung function (Frisancho, 1969; Mueller et al., 1978b; Whittaker, 1992: but see Brutsaert et al., 1999; Tarazona‐Santos et al., 2000) and so may improve work efficiency rather than impacting growth. Finally, others have suggested that only the most nutritionally‐stressed Andean populations show increased chest growth (Pawson and Huicho, 2010; Pawson et al., 2001), but the relationship between nutritional status, chest size and linear growth remains to be directly tested.

Alternative explanations for our findings include the possibility that differences in limb and body proportions are due entirely to genetic factors. This would seem unlikely given that, while worldwide population differences in body size and proportions are thought to include a genetic component (Eveleth and Tanner, 1990), it probably accounts for only a small proportion of global variation in relative sitting height (head‐trunk height relative to stature: ∼3.6%: Bogin et al., 2001). Furthermore, differences in relative limb proportions are greater between populations of European vs. African or Australian ancestry than between those of European vs. Asian (including native American) ancestry (Bogin et al., 2001; Feldesman and Fountain, 1996; Stinson, 2009), and most migrants to Peru have come from Europe (Bigham et al., 2008; Sanchez et al., 2010).

Whether Andean ancestry is associated with genetic differences in body proportions has not been extensively studied. Adaptation to cold climates (e.g., Trinkaus, 1981) or moving over steep terrain (e.g., Higgins and Ruff, 2011) has been proposed to result in relatively shorter limbs and especially distal limb segments (tibia), but evidence for such relationships among highland Andeans is lacking. The Multinational Andean Genetic and Health Program studies of north Chilean Aymara‐speakers gave contradictory results regarding the relationship between ancestry, altitude, and body size and proportions (Mueller et al., 1978a; Palomino et al., 1978). Stinson (2009) investigated stature and relative sitting height among poorer rural and wealthier urban Bolivian children aged 8–13. Although ancestry, nutrition, SES and time spent at altitude were related and difficult to separate, she suggested that Bolivian ancestry (parents born in Bolivia) and time at altitude might both be associated with relatively shorter legs within the wealthier urban sample, although the relative influences of multiple factors could not be assessed. Variation with ancestry was not investigated within her rural sample. The relationships between these different environmental characteristics, ancestry, and body size and proportions thus also remain to be elucidated.

The lack of any relationship between growth measures and parental surnames among lowland children in our study suggests that the variation in body size and proportions with inferred ancestry among highland children may result from gene‐environment interactions, although this interpretation has to be cautious. Acculturation, associated with the tendency to Hispanicize indigenous surnames (Schull and Rothhammer, 1977), is greater in lowland communities and the lowland sample likely represents a greater mix of different indigenous populations than the highland sample, since the settlement comprises migrants from various rural highland regions of Peru (Checkley et al., 2002), as well as a small contingent from the coast and Amazon. Although the majority of children in the lowland sample were likely to have been of largely highland Andean ancestry, we can neither confirm this objectively nor exclude a genetic difference in body proportions between the highland and lowland samples that is not environment‐dependent. Another potential explanation for our results among highland children is that parental surnames are related not only to ancestry but also to other environmental variables like SES, which the available variables may not have controlled for adequately. This possibility also requires further investigation.

Whether high‐altitude neonates show any relationship between body size and proportions and Andean ancestry is unclear. While the effects of Andean ancestry on birth weight at altitude are consistent across studies, few consider differences in other neonatal anthropometry. Birth length, head circumference, limb lengths, skinfold thicknesses and weight are generally reduced at altitude (Giussani et al., 2001; Haas, 1976; Haas et al., 1977, 1980; McClung, 1969; Mortola et al., 2000; Soria et al., 2013). Haas et al. (1980) suggested that the decrease in crown‐heel length with altitude was greater among neonates of European compared with Andean ancestry, though Soría et al. (2013) did not replicate this finding.

Other studies demonstrate a positive association between birth weight and adult height and weight (Eide et al., 2005; Sørensen et al., 1999; Yliharsila et al., 2007), so the apparent loss of this early growth advantage among children of Andean ancestry at altitude requires explanation. It may reflect the fact that higher birth weight at altitude among Andeans results from maternal physiological adaptations, including greater maternal uterine artery blood flow, which is augmented in the third trimester by ∼36% (Julian et al., 2008; but see Postigo et al., 2009; Wilson et al., 2007; Zamudio et al., 2007), and increased resting respiratory rate (Vargas et al., 2007) compared with non‐Andean mothers.

Higher birth weight is also positively associated with neonatal survival (except at extremely high birthweight: Karn and Penrose, 1951; Wilcox and Russell, 1983), therefore the greater birth weight associated with Andean ancestry may confer benefits in early postnatal survival at altitude rather than growth. Such a survival advantage has been shown for Tibetan (high‐altitude native) vs. Han Chinese (recent migrants) at high altitude (Moore et al., 2001). Greater fat mass rather than lean mass may be key to early infant survival at altitude (Wiley, 1994), and newborns at altitude in Denver, Colorado have reduced subcutaneous fat but not lean mass, head circumference or femur length (Galan et al., 2001). This may suggest that fat mass is disproportionately affected by high‐altitude gestation. In a Bolivian high‐altitude sample, surname–inferred Andean ancestry was associated with greater birth weight but no difference in length compared with European ancestry, which would be consistent with greater fat accrual among Andean neonates at altitude, although direct data on body composition were unavailable and differences in ponderal index insignificant (Soria et al., 2013). If the greater birth weight of highland Andean infants reflects greater fat rather than lean mass, this could explain the positive effects of Andean ancestry on birth weight but not postnatal height and lower limb length in the highlands.

Strengths of our study include the detailed anthropometry, including limb segment lengths, which allowed us to investigate in greater detail the relationship between body proportions and ancestry than is possible in many studies. Furthermore, while a large number of studies have considered ancestry in relation to neonatal anthropometry, few have considered the relationship between ancestry and body size or proportions in infants and children. Unfortunately, we were unable to explore whether the associations between anthropometry and ancestry might reflect trade‐offs with chest growth, or whether they persist into adulthood. Investigating these problems in the future would help to shed light on the nature and cause of differences in body size and proportions by ancestry in the Andean highlands. Furthermore, our data were cross‐sectional, and therefore we were unable to assess longitudinal differences in body size, proportions, and growth patterns according to ancestry in our samples.

The use of genetic data rather than surnames (which are less accurate markers of ancestry) would also serve to elucidate the nature of links between ancestry and body size and proportions. While Chakraborty et al. (1989) showed that the number of indigenous surnames correlated with genetic markers of Andean ancestry, their study considered north Chilean and Bolivian populations only, reported a limited range of markers (as expected for a study of its date), and showed that even those with no indigenous parental surnames had a significant proportion of Andean admixture. Those with no indigenous surnames had on average 64% “native American” loci, compared with 85% for those with 1–2 indigenous names, and 89% for those with 3–4. A more recent study of highland and lowland Bolivian women similarly showed a good correspondence between surnames and genetic indigenous or European ancestry using over 100 Ancestry Informative Markers (AIMs) (Julian et al., 2009). While the relationship has not been directly tested in our study samples, it is likely that surnames provide a reasonable proxy for ancestry in our analyses.

In conclusion, greater Andean ancestry (inferred from numbers of indigenous parental surnames) was negatively associated with anthropometry among highland, but not lowland, Peruvian children. In particular, highland children with more indigenous parental surnames had significantly shorter tibia length (absolutely and relative to trunk length), lower limb length, ulna length, and stature. Within the highland sample, altitude also showed a negative relationship with anthropometry *z* scores, and the ancestry and altitude associations with anthropometry in the highland sample were independent. The results suggested that the effect of indigenous Andean ancestry on increasing birth weight at altitude as demonstrated in other studies does not translate into larger postnatal body size. Previously proposed trade‐offs between stature and chest size, contingent on Andean ancestry and exposure to hypoxia, may explain the pattern of results, but we were unable to test this hypothesis directly.

## Supporting information

Supporting Information
