# Supplementary material for: Surname‐Inferred andean ancestry is associated with child stature and limb lengths at high altitude in Peru, but not at sea level
Source: Am J Hum Biol. 2015 May 11;27(6):798–806. doi: 10.1002/ajhb.22725 (PMC4607539; doi:10.1002/ajhb.22725)
Supplement: Supplementary file 1 — Supporting Information [file AJHB-27-798-s001.doc]

**Supporting Table 1. Exclusions from the dataset of children aged 6 months to 8.5 years.**

|  | **Highland** | | **Lowland** | |
| --- | --- | --- | --- | --- |
|  | **n** | **% of total** | **n** | **% of total** |
| Total | 163 | 100 | 186 | 100 |
| Excluded: missing surnames | 27 | 18 | 14 | 8 |
| Excluded: missing confounding variables | 3 | 2 | 3 | 2 |
| **Final sample** | **133** | **80** | **169** | **90** |

**Supporting Table 2. Full results of regression models of anthropometry z scores on the number of indigenous parental surnames and potential confounding variables among highland Peruvian children.**

| **Measurement**  **z score** | **Head-trunk length z score ^1^** | | **Altitude (km)** | | **Mother’s age at child’s birth** | | **Mother’s education ^2^** | | | | **N of indigenous parental surnames** | | **Model p** | **Adjusted R^2^** |
| --- | --- | --- | --- | --- | --- | --- | --- | --- | --- | --- | --- | --- | --- | --- |
|  | **B** | **p** | **B** | **p** | **B** | **p** | **1** | | **3** | | **B** | **p** |  |  |
|  |  |  |  |  |  |  | **B** | **p** | **B** | **p** |  |  |  |  |
| Head circumference |  |  |  |  | **0.02** | **0.04** | -0.10 | 0.6 | **0.35** | **0.04** | -0.11 | 0.08 | **0.02** | 0.06 |
| Stature |  |  | **-0.062** | **0.001** |  |  | **-0.136** | **0.03** | 0.06 | 0.7 | **-0.14** | **0.01** | **<0.001** | 0.14 |
| Head-trunk height |  |  |  |  |  |  | -0.25 | 0.2 | 0.28 | 0.08 | -0.12 | 0.06 | **0.02** | 0.06 |
| Upper limb length |  |  | **-0.061** | **0.001** |  |  | **-0.51** | **0.003** | -0.02 | 0.9 | -0.09 | 0.1 | **<0.001** | 0.15 |
| Ulna length |  |  | **-0.072** | **<0.001** |  |  | **-0.49** | **0.002** | -0.01 | 1.0 | **-0.12** | **0.03** | **<0.001** | 0.21 |
| Hand length |  |  |  |  |  |  | **-0.56** | **0.003** | 0.04 | 0.8 | -0.09 | 0.2 | **0.008** | 0.09 |
| Lower limb length |  |  | **-0.066** | **0.001** |  |  |  |  |  |  | **-0.16** | **0.01** | **<0.001** | 0.14 |
| Tibia length |  |  | **-0.065** | **<0.001** | **-0.02** | **0.03** |  |  |  |  | **-0.18** | **0.002** | **<0.001** | 0.21 |
| Foot length |  |  | -0.037 | 0.07 |  |  | **-0.39** | **0.03** | -0.04 | 0.8 | -0.09 | 0.2 | **0.05** | 0.05 |
| Relative upper limb length | **0.55** | **<0.001** | **-0.040** | **0.003** |  |  |  |  |  |  | -0.05 | 0.5 | **<0.001** | 0.47 |
| Relative ulna length | **0.50** | **<0.001** | **-0.049** | **0.001** |  |  |  |  |  |  | -0.08 | 0.09 | **<0.001** | 0.46 |
| Relative lower limb length | **0.38** | **<0.001** | **-0.057** | **0.002** |  |  |  |  |  |  | **-0.12** | **0.04** | **<0.001** | 0.26 |
| Relative tibia length | **0.45** | **<0.001** | **-0.049** | **0.001** |  |  |  |  |  |  | **-0.13** | **0.005** | **<0.001** | 0.43 |

Birth order omitted as not significant in any model. **Bold** indicates p<0.05.

^1^ Head-trunk height z score included only in relative limb length models. For other variables, blank cells denote variable excluded from model as p<0.1.

**^2^** Mother’s education: 1 = None; 2 (reference) = Incomplete primary; 3 = Complete primary or higher.

**Supporting Table 3. Results of regression models of anthropometry z scores on the number of indigenous parental surnames and potential confounding variables among lowland Peruvian children.**

| **Measurement**  **z score** | **Head-trunk length z score ^1^** | | **Birth order** | | | | | | **Mother’s education ^2^** | | | | | | **N of indigenous parental surnames** | | **Model**  **p** | **Adjusted R^2^** |
| --- | --- | --- | --- | --- | --- | --- | --- | --- | --- | --- | --- | --- | --- | --- | --- | --- | --- | --- |
|  | **B** | **p** | **2** | | **3** | | **4+** | | **1** | | **3** | | **4** | | **B** | **p** |  |  |
|  |  |  | **B** | **p** | **B** | **p** | **B** | **p** | **B** | **p** | **B** | **p** | **B** | **p** |  |  |  |  |
| Head circumference |  |  |  |  |  |  |  |  |  |  |  |  |  |  | -0.03 | 0.7 | 0.7 | 0 |
| Stature |  |  | -0.06 | 0.6 | -0.22 | 0.2 | **-0.86** | **<0.001** |  |  |  |  |  |  | 0.04 | 0.5 | **0.001** | 0.09 |
| Head-trunk height |  |  | 0.0 | 1.0 | -0.22 | 0.3 | **-0.85** | **0.002** |  |  |  |  |  |  | 0.08 | 0.3 | **0.02** | 0.05 |
| Upper limb length |  |  | -0.21 | 0.1 | -0.22 | 0.2 | **-0.75** | **<0.001** |  |  |  |  |  |  | 0.02 | 0.7 | **0.007** | 0.07 |
| Ulna length |  |  |  |  |  |  |  |  | -0.19 | 0.2 | 0.29 | 0.08 | **0.38** | **0.005** | 0.01 | 0.9 | **0.003** | 0.08 |
| Hand length |  |  |  |  |  |  |  |  | **-0.31** | **0.06** | 0.21 | 0.3 | 0.21 | 0.2 | -0.03 | 0.7 | **0.04** | 0.04 |
| Lower limb length |  |  |  |  |  |  |  |  | **-0.39** | **0.003** | 0.02 | 0.9 | **0.26** | **0.05** | -0.02 | 0.7 | **0.001** | 0.09 |
| Tibia length |  |  | -0.08 | 0.4 | -0.11 | 0.5 | **-0.65** | **0.001** |  |  |  |  |  |  | 0.03 | 0.6 | **0.02** | 0.05 |
| Foot length |  |  |  |  |  |  |  |  |  |  |  |  |  |  | -0.02 | 0.7 | 0.7 | 0 |
| Relative upper limb length | **0.39** | **<0.001** |  |  |  |  |  |  | -0.17 | 0.2 | 0.11 | 0.5 | **0.29** | **0.02** | -0.01 | 0.8 | **<0.001** | 0.29 |
| Relative ulna length | **0.36** | **<0.001** |  |  |  |  |  |  | -0.20 | 0.09 | 0.11 | 0.45 | **0.33** | **0.007** | -0.02 | 0.7 | **<0.001** | 0.27 |
| Relative lower limb length | **0.21** | **<0.001** |  |  |  |  |  |  | **-0.38** | **0.003** | -0.08 | 0.6 | 0.23 | 0.07 | -0.04 | 0.5 | **<0.001** | 0.16 |
| Relative tibia length | **0.32** | **<0.001** |  |  |  |  |  |  | **-0.24** | **0.04** | -0.17 | 0.2 | 0.19 | 0.09 | -0.004 | 0.9 | **<0.001** | 0.23 |

**Bold** indicates p<0.05.

**^1^** Head-trunk height z score included only in relative limb length models. For other variables, blank cells denote variable excluded from model as p<0.1.

**^2^** Maternal education: 1 = Primary (complete or incomplete) or incomplete secondary; 2 (reference) = Complete secondary; 3 = Incomplete post-secondary; 4 = complete post-secondary.
